# From shadowing to active learning: exploring the impact of supervised teaching clinics on gynecology education

**DOI:** 10.3389/fmed.2024.1498393

**Published:** 2025-01-07

**Authors:** Sangsang Ren, Hong Zhan, Asanga Fernando, Xiangrong Xu, Weiguo Lu

**Affiliations:** ^1^College of Education, Zhejiang University, Hangzhou, China; ^2^Department of Gynecology, Women’s Hospital, Zhejiang University School of Medicine, Hangzhou, China; ^3^Departments of Medical Oncology, Liaison Psychiatry and Simulation and Clinical Skills, St George’s University Hospitals NHS Foundation Trust, London, United Kingdom; ^4^St George’s, University of London, London, United Kingdom; ^5^Department of Education, Women’s Hospital, Zhejiang University School of Medicine, Hangzhou, China; ^6^Department of Gynecologic Oncology, Women’s Hospital, Zhejiang University School of Medicine, Hangzhou, China

**Keywords:** supervised teaching clinic, medical education, competence-based education, professional development, self-efficacy

## Abstract

**Background:**

Supervised Teaching Clinics (STCs) have emerged as an innovative approach to medical education, particularly in specialties like gynecology, where hands-on experience is crucial. Traditional clinical rotations often leave students in passive roles, limiting their active participation and the development of essential clinical skills.

**Aim:**

This study aimed to evaluate the impact of STCs on the clinical competencies and professional development of medical students within a gynecological clinic, comparing the outcomes with those of traditional clinic shadowing.

**Methods:**

A total of 144 fifth-year medical students were randomly assigned to either a control group, which participated in traditional clinic shadowing, or an STC group, which engaged in both shadowing and supervised teaching activities. The study utilized the Generalized Self-Efficacy Scale (GSES) and the mini-Clinical Evaluation Exercise (mini-CEX) to assess clinical performance. Feedback was also collected from students, tutors, and patients to gage satisfaction and perceived effectiveness.

**Results:**

Students in the STC group demonstrated significantly higher improvements in clinical skills, particularly in medical interviewing, counseling, and overall clinical competence, compared to the control group. The STC group also reported greater satisfaction with their learning experience, citing enhanced confidence and a deeper understanding of gynecological practice. Tutors and patients provided positive feedback, noting the STC’s role in fostering effective student-patient interactions and comprehensive learning.

**Conclusion:**

The structured design of the STC, with a focus on goal direction, relationships, and supporting services, significantly improved educational outcomes in gynecology. By fostering active learning and delivering constructive feedback, STCs effectively enhance students’ clinical competencies and professional development. The study suggests that integrating STCs into traditional clinical education models could substantially optimize medical training.

## 1 Introduction

Over the last decade, competence-based medical education (CBME) has been advocated in medical teaching that accentuates medical knowledge, interpersonal and communication skills, and professional identity (1). Despite emphasis on ‘student-centered’ education, undergraduate medical students, unlike medical residents, often find themselves in passive, observational roles during clinical rotations. As a result, students have limited opportunities to reflect on their professional identity as future doctors and may later struggle to interact effectively with patients (2). This limited engagement can hinder the development of critical reflection, a cornerstone of effective medical practice. Critical reflection (3, 4) is a central tenet of good medical practice and should be cultivated early in medical students’ training for their careers. Most students report the desire to be supervised by their tutors during consultations or clinical examinations with autonomy and protection (5). To address this gap, there is a growing need for educational environments that provide students with active learning opportunities, allowing them to integrate theoretical knowledge with practical skills in a real-world context.

Teaching at the bedside or outpatient clinics is at the heart of medical education and provides essential clinical training (6). Compared to inpatient clinical work, outpatient clinical work is less time-consuming but provides different and unique opportunities to listen to and examine patients including their ideas, concerns and expectations. Thus, teaching in a clinic is an essential and irreplaceable part of education for medical students (7).

A supervised teaching clinic (STC) represents a strategic approach to fostering such a safe and supportive environment, particularly within the outpatient setting. Rather than the routine clinic shadowing, an STC is defined as a student-led consultation of real patients under the supervision of experienced tutors. This approach ensures that patients’ rights are always respected during teaching and learning activities.

This study evaluates the implementation of an STC within a gynecological clinic, focusing on its effectiveness in enhancing students’ clinical competencies and professional development. The analysis is framed around three key dimensions: goal direction, relationships, and supporting services, which are critical to creating a conducive learning environment.

## 2 Materials and methods

### 2.1 Routine gynecology clinic rotation

**Figure 1:**
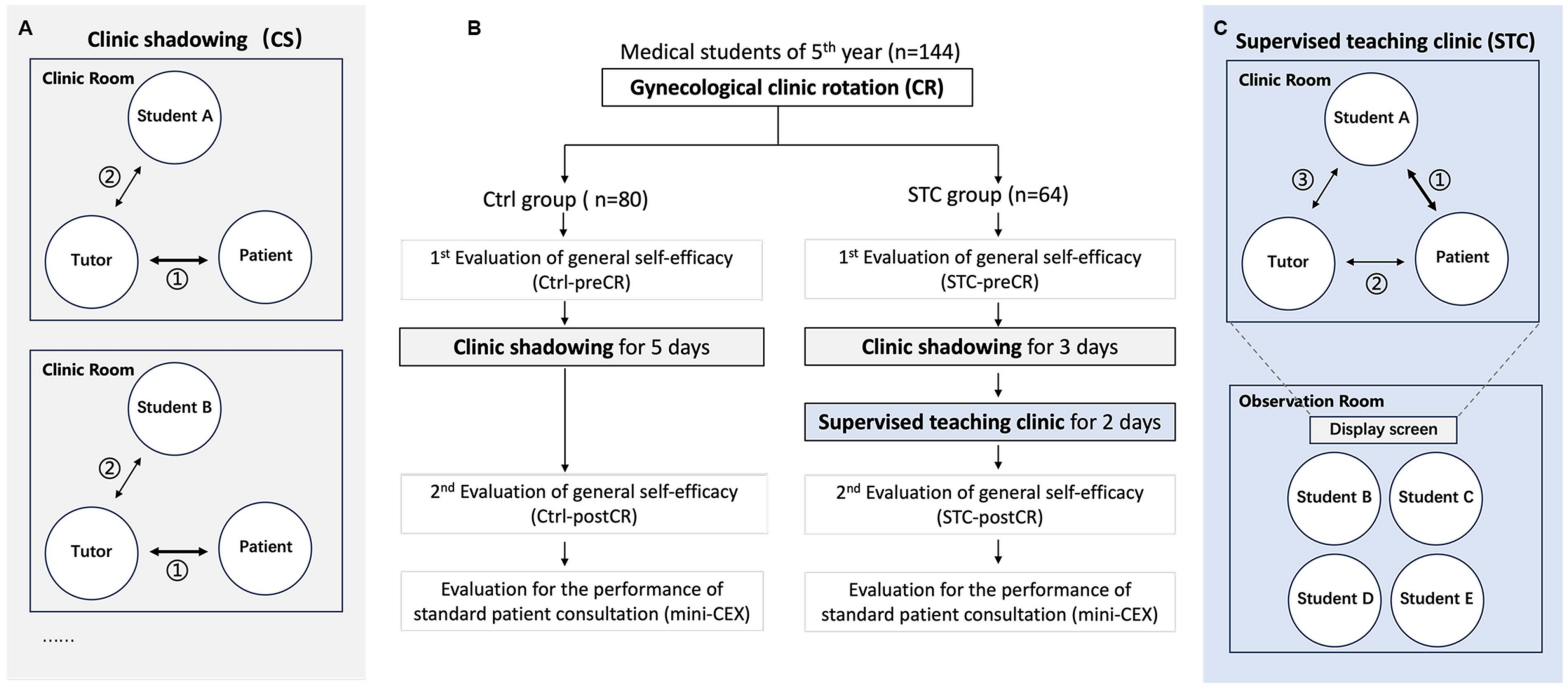
Overview and flow diagram of the study design. **(A)** The gray box on the left represents the traditional clinic rotation (clinic shadow), where the primary interaction occurs between the tutor and the patient. After the session, the tutor may briefly discuss the case with the student. **(B)** The diagram outlines the overall clinic rotation process. **(C)** The blue box on the right represents the supervised teaching clinic (STC), where the primary interaction occurs between the student and the patient. The tutor may interact with the patient afterward or as needed, followed by a feedback session between the tutor and the student.

This study was carried out at Zhejiang University. Before participating in the STC, all students completed a clerkship involving theoretical courses including Internal Medicine, Surgery, Obstetrics and Gynecology, Paediatrics, and etc. As students had completed a gynecological inpatient rotation, they were familiar with the diagnosis and treatment of common gynecological conditions. They had also been trained to perform standard physical examinations. In routine gynecology clinic rotations, 5th year medical students spend a 5-day clerkship for an outpatient clinic shadowing, where they are supposed to observe their tutors’ consultations with various gynecologic patients. During this clinical shadowing, the primary focus is on the consultation between the attending physician and the patient, with the student primarily observing, as is typical in shadowing experiences. In some cases, they may be encouraged to perform simple procedures (Figure 1A). At the end of the gynecological rotation, students complete a consultation with a standardized patient (SP) and perform a physical examination of a model. Tutors would evaluate all the consultations using the Mini Clinical Exercise evaluation (mini-CEX) (8) with unbiased scores.

### 2.2 Participants

A total of 144 rotation-batched medical students were divided by stratified random sampling (Supplementary Table S1) into (i) a control (Ctrl) group (*n* = 80; 35 males and 45 females) and (ii) a supervised teaching clinic (STC) group (*n* = 64; 30 males, 34 females) (Figure 1B). The STC group was further divided into subgroups containing 4 students each. The Ctrl group completed the 5 days’ clinic shadowing, while the STC group spent 3 days for clinic shadowing and 2 days for performing the STC during the rotation. Six gynecologic tutors (associate chief gynecologist or above) with at least 3 years of experience in clinical teaching oversaw the STC. We also recruited 97 gynecologic patients without emergent conditions or mental diseases in STC (Supplementary Table S2).

### 2.3 STC design

During the STC, a student performed a consultation with a real patient under the supervision of the tutor. The STC involved three steps: First, a student independently interviewed a RP for consultation and performed a physical examination (including pelvic examination) if necessary. Second, the tutor made supplements or corrections (if necessary) to provide the patient with proper diagnosis and treatment. Third, after the patient left, the tutor commented on the student’s performance with proper feedback. During the STC, the consultation by the medical student is consider the most critical part, and the tutor tried not to interrupt the consultation and only provided minor suggestions if necessary. At the same time, students in the same subgroup sat in another room and observed the whole process (except the pelvic examination). Each student in the STC group participated in the consultation involving the real patients (Figure 1C). Written informed consent was obtained from each participant.

### 2.4 Outcome measures

**Figure 2:**
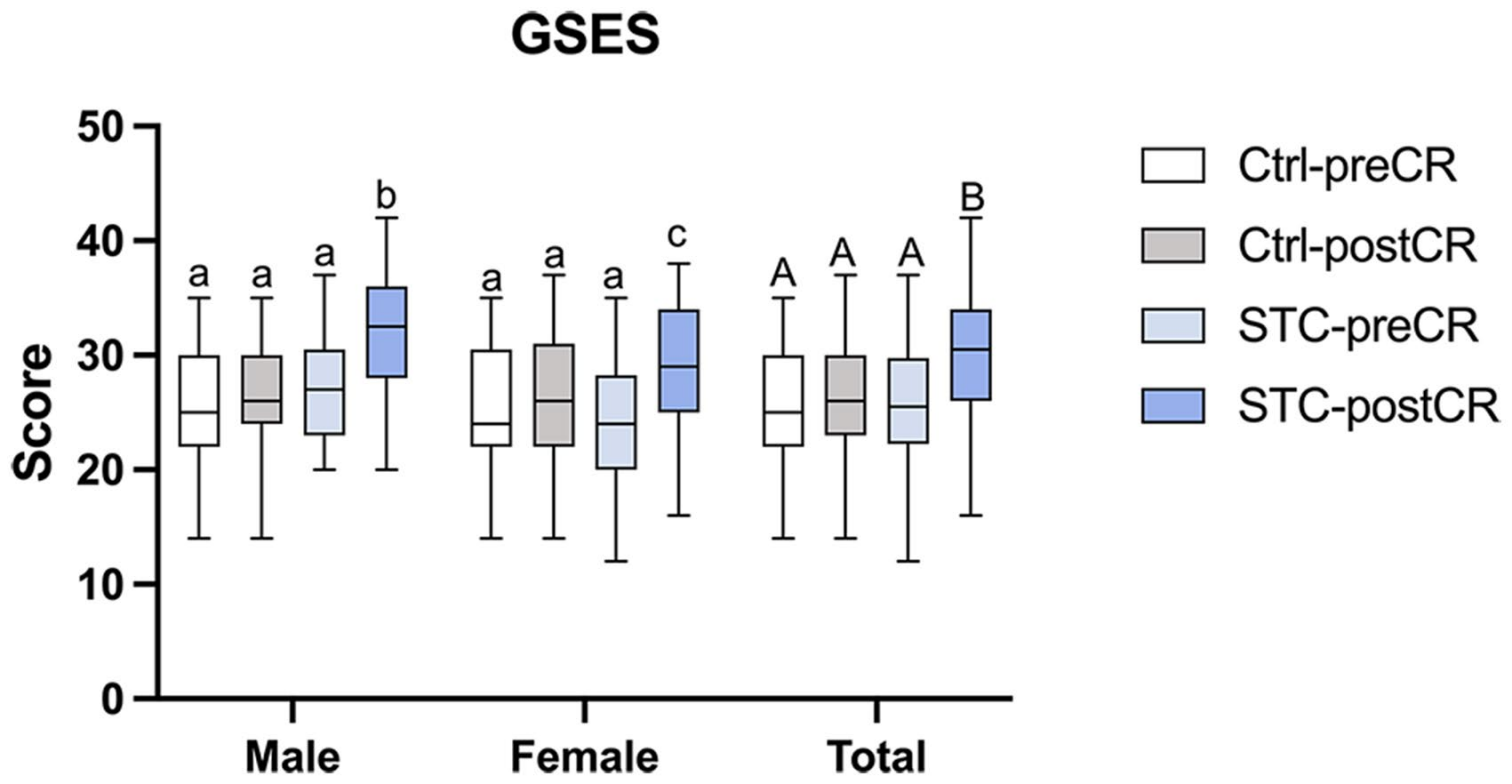
Students’ general self-efficacy before and after clinic rotation (CR). Results are expressed as the box plots. Lines represent min to max intervals. Higher numbers represent better scores. Values without a common letter are significantly different (*p* < 0.05). GSES = general self-efficacy scale; CR = clinic rotation; STC = supervised teaching clinic; Ctrl-preCR = students’ GSES in the control group before clinic rotation (white box); Ctrl-postCR = students’ GSES in the control group after clinic rotation (gray box); STC-preCR = students’ GSES in the STC group before clinic rotation (light blue box); STC-postCR = students’ GSES in the STC group after clinic rotation (blue box).

**Figure 3:**
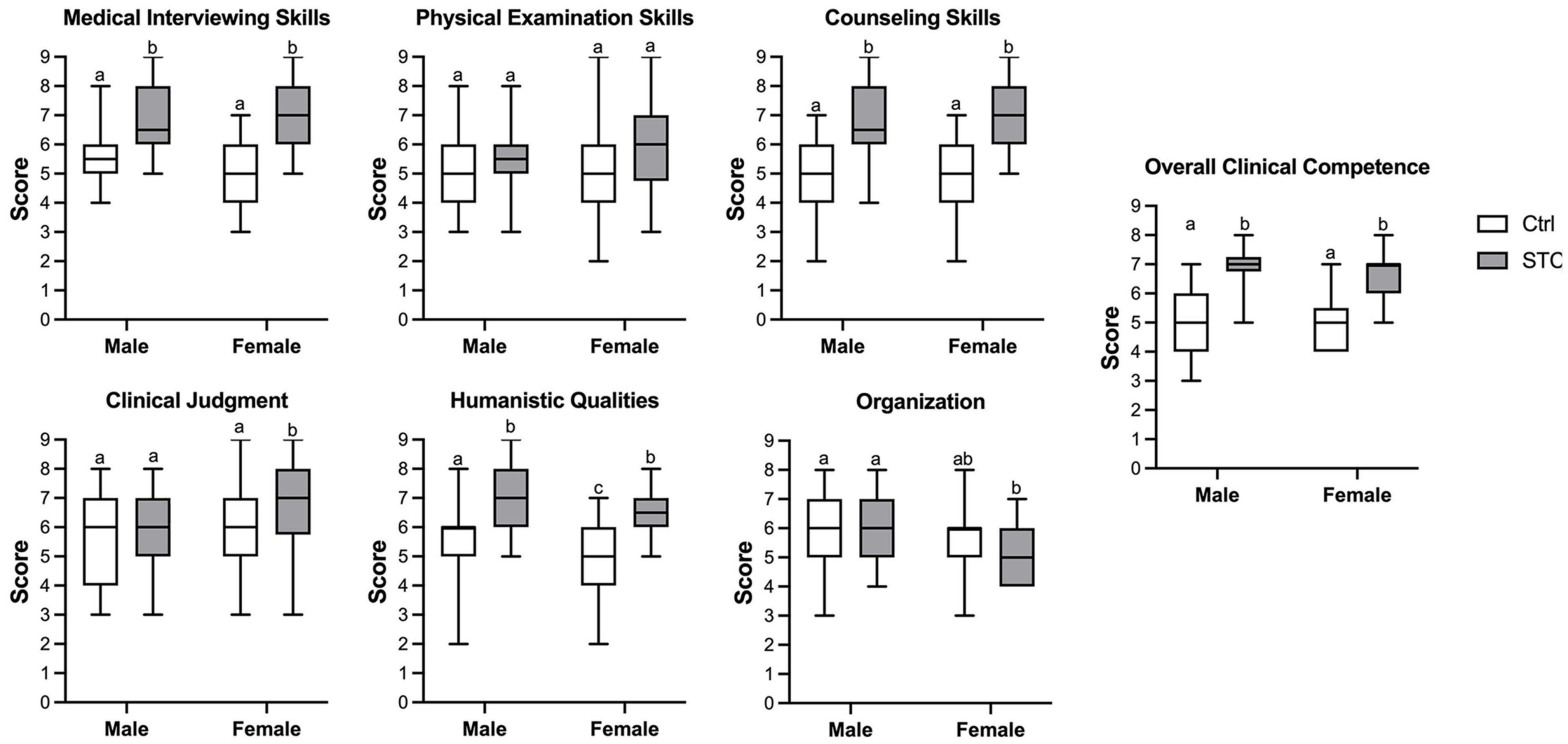
The performance of students’ consultation with standard patients. All the students’ performance of consultations with standard patients are evaluated independently completed the Mini Clinical Exercise evaluation (mini-CEX). Results are expressed as the box plots. Lines represent min to max intervals. Higher numbers represent better scores. Values without a common letter are significantly different (*p* < 0.05). Ctrl = control group; STC = the supervised teaching clinic group.

Students’ confidence and optimistic self-beliefs were evaluated using the Generalized Self-Efficacy Scale (GSES) (9). Students in both control group and STC group completed the GSES before and after the clinic rotation. All the students’ performance of consultations are evaluated by the Ctrl and STC groups independently completed the mini-CEX of consultation with an SP (Figure 1B). After participating in the STC, students completed an 8-item questionnaire to express their experiences and needs. The patients completed an 8-item questionnaire to report their perceptions of the consultation. Responses were measured by a 5-point Likert scale, from 1 indicating “strongly disagree” to 5 indicating “strongly agree.” Incomplete questionnaires (with no response to one or more of the eight questions) were not counted. Tutors were encouraged to write down their experiences and feelings during the STC. All data were collected and analyzed (see Figures 2, 3).

### 2.5 Statistical analysis

Continuous variables are reported as the mean ± standard deviation. Data were analyzed by two-way analysis of variance (ANOVA) followed by Tukey’s *post hoc* tests. Comparisons between groups were made using the paired student’s *t*-test for continuous variables. Results with *p* < 0.05 were considered statistically significant. Statistical analysis was conducted using online GraphPad Software.

## 3 Results

### 3.1 Students’ GSES scores

After the STC training, students’ general self-efficacy improved. There was no significant difference between the Ctrl and STC groups before clinic rotation (Ctrl-preCR *vs.* STC-preCR), and no significant difference before and after the clinic rotation in Ctrl group (Ctrl-preCR *vs.* Ctrl-postCR). However, the GSES scores increased after the STC training (STC-preCR *vs.* STC-postCR). Interestingly, the increase in GSES scores was more pronounced among male medical students compared to female students in STC group (Figure 2).

### 3.2 Students’ performance of consultation after the gynecology clinic rotation

The performance of consultation with the SPs were compared between the Ctrl and STC groups at the end of the clinic rotation (Ctrl *vs.* STC). Both male and female students in the STC group scored higher on medical interviewing skills, counseling skills, humanistic qualities, and overall clinical competence. Furthermore, female students seem to get improvement in the clinical judgment, but not organization, rather than male students. There was no significant difference in physical examination skills, clinical judgment and organization scores between Ctrl and the STC group (Figure 3).

### 3.3 Feedback on the STC

All students in the STC group (*n* = 64) completed the questionnaire. The mean (SD) scores for the 8 items are as follows: “STC is necessary for clerkship” 4.92 (0.27); “STC meets the internship expectations” 4.84 (0.44); “STC is systematic and logical” 4.77 (0.52); “STC improves your clinical practice skills” 4.34 (0.94); “STC improves your clinical judgment” 4.64 (0.65); “STC improves your counseling skills” 4.86 (0.35); “You are satisfied with the overall experience” 4.80 (0.51); and “You expect more opportunities of STC” 4.81 (0.46) (Table 1).

**Table 1:** Feedback responses of STC from students.

Feedback questions	Reponses (score)					Mean score ± SD
	Strongly disagree (1)	Disagree (2)	Neutral (3)	Agree (4)	Strongly agree (5)	
Students (*n* = 64)						
1. Necessary for clerkship	–	–	–	5	59	4.92 ± 0.27
2. Meeting the training expectations	–	–	2	6	56	4.84 ± 0.44
3. Systematic and logical	–	–	3	9	52	4.77 ± 0.52
4. Improving your practice skills	–	5	6	15	38	4.34 ± 0.94
5. Improving your clinical judgment	–	–	6	11	47	4.64 ± 0.65
6. Improving your counseling skill	–	–	–	9	55	4.86 ± 0.35
7. Satisfaction with overall experience	–	–	3	7	54	4.80 ± 0.51
8. Expectation of more STC chances	–	–	2	8	54	4.81 ± 0.46

Feedback on the STC was also collected from six tutors. They agreed with the items as follows: “Necessary for clerkship” (*n* = 5), “Helpful and meaningful for undergraduates” (*n* = 4), “A challenge for both students and tutors” (*n* = 3), “Mini-CEX is great to evaluate students’ performance” (*n* = 3), “Surprised by patients’ high satisfaction” (*n* = 2), and “The consultation is time consuming” (*n* = 2) (Table 2).

**Table 2:** Interview of tutors’ experiences of STC.

Subjective opinions collected through interviews	*N* = 6
Necessary for clerkship	5
Helpful and meaningful for undergraduates	4
A challenge for both students and tutors	3
Mini-CEX is great to evaluate students’ performance	3
Surprised of patients’ high satisfaction	2
Inefficiency	2

A total of 97 patient surveys were received and analyzed. All patients felt satisfied with the STC experience at different levels. The mean (SD) scores for the 8 questions were as follows: “Your doctor is qualified” 4.91 (0.29); “You received appropriate treatment” 4.88 (0.33); “It was effective counseling “4.96 (0.20); “Your doctor complied with rigorous medical ethics” 4.99 (0.10); “Your privacy was well-protected” 4.87 (0.34); “You were in a safe and comfort environment during the consultation” 4.94 (0.34); “You were satisfied with the overall experience” 4.94 (0.34); and “You would recommend STC to others” 4.87 (0.34) (Table 3).

**Table 3:** Feedback responses of STC from patients.

Feedback questions	Reponses (score)					Mean score ± SD
	Strongly disagree (1)	Disagree (2)	Neutral (3)	Agree (4)	Strongly agree (5)	
Patients (*n* = 97)						
1. Qualified doctor	–	–	–	9	88	4.91 ± 0.29
2. Appropriate treatment	–	–	–	12	85	4.88 ± 0.33
3. Effective counseling	–	–	–	4	93	4.96 ± 0.20
4. Rigorous medical ethics	–	–	–	1	96	4.99 ± 0.10
5. Well privacy protection	–	–	–	13	84	4.87 ± 0.34
6. Safe and comfort Environment	–	–	–	2	95	4.94 ± 0.43
7. Satisfaction of overall experience	–	–	–	2	95	4.94 ± 0.43
8. Recommendation for others	–	–	–	13	84	4.87 ± 0.34

## 4 Discussion

**Figure 4:**
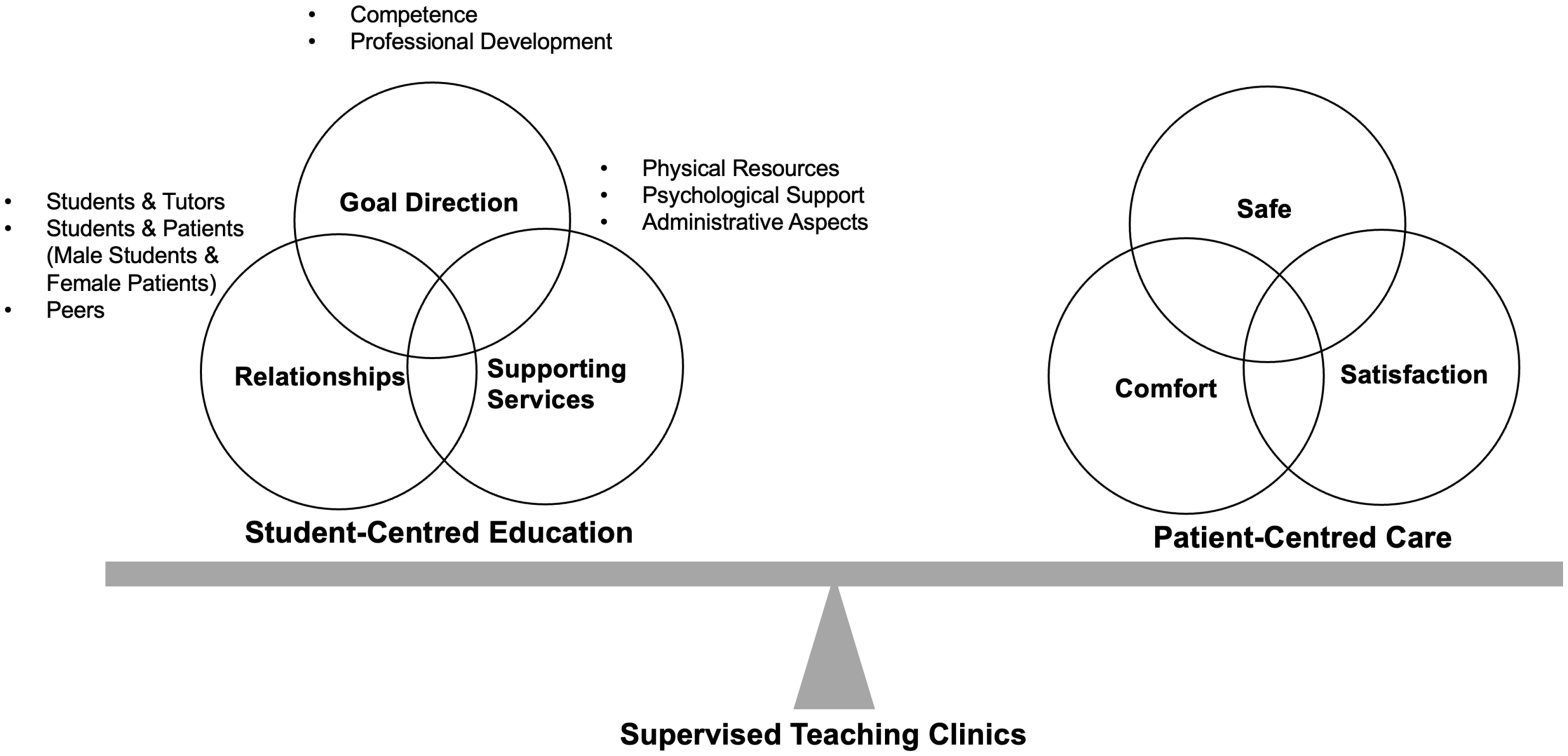
Balancing Student-Centered Education and Patient-Centered Care in the Supervised Teaching Clinic (STC) Model. The structured design of the supervised teaching clinic (STC) model effectively balances student-centered education with patient-centered care. The model is built upon three key pillars: goal direction, relationships, and supporting services.

A well-established theoretical framework for describing educational environments puts forward three domains as critical to the quality of human environments: goal direction or content of education, relationships, and supporting services (10). It was also applicable to the educational context (11), serving as a framework to explore the needs, expectations and experiences of medical students regarding their learning during the STC. These findings suggest that the structured design of the STC model effectively balances student-centered education with patient-centered care (Figure 4).

### 4.1 Goal direction

The implementation of the STC in this study aligns strongly with the dimension of goal direction, a fundamental aspect of creating a conducive learning environment. In the context of medical education, goal direction refers to the clarity of educational objectives and the systematic approach toward achieving them (12). Within the STC, students were not merely passive observers but active participants in patient care, a shift that is essential for their professional development.

The STC provided a structured platform where students could set and pursue clear, tangible goals. This focus on goal-oriented tasks allowed students to progressively build their competencies in a controlled environment, with the opportunity to receive immediate feedback and make necessary adjustments. The observed improvement in students’ GSES after participating in the STC is a testament to the effectiveness of this approach. Self-efficacy, a crucial factor in professional development, is enhanced when learners can achieve small, incremental goals, leading to greater confidence and autonomy in their future practice (13).

The STC’s structure also prompted students to reflect on their professional identity. Allowing students to interview patients independently results in psychological insights and deeper learning reflections, emphasizing the concepts of communication, humanity, and empathy in the learning process that goes beyond medical theoretical knowledge and clinical skills exercises. Students reported that the STC was necessary for developing clinical judgment, practice skills, and counseling skills during their clerkship. By engaging in real patient interactions, students might be able to visualize their future roles as healthcare providers, a critical aspect of their professional identity formation. This aligns with the broader goals of CBME, where the emphasis is on not just acquiring knowledge, but also on developing the attitudes and behaviors necessary for professional practice. The ability to independently conduct patient interviews and physical exams, while under supervision, provided students with a unique opportunity to integrate their theoretical knowledge with practical skills, thereby solidifying their understanding of their professional responsibilities.

The goal direction within the STC was further reinforced by the use of structured evaluation tools like the mini-CEX. Mini-CEX is an appropriate tool for formative evaluation during clinical competence. Mini-CEX has been shown to enhance student-patient interactions, leading to improved care services, while also strengthening the interactions between students and evaluators during the assessment of clinical skills (8, 14). This tool provided a clear framework for tracking students’ progress and ensured feedback was focused and actionable. The systematic approach to evaluation helped students to identify areas for improvement and monitor their progress, contributing to a more effective learning experience.

### 4.2 Relationships and interpersonal skills

The relational dimension of the STC cannot be overstated. Relationships, both between students and tutors, as well as between students and patients, play a pivotal role in the learning process. In traditional clinical settings, the hierarchical nature of the student-tutor relationship often limits the extent to which students can actively engage in patient care. However, the STC model employed in this study sought to redefine these relationships by fostering a more collaborative learning environment.

Achieving a balance between autonomy and supervision is often challenging, particularly in the context of medical education (15). Within the STC, the balance was characterized during the tutor-student relationship. Tutors provided guidance and support without overshadowing the students’ active participation in patient consultations. This balance is crucial in medical education, as it allows students to develop confidence in their clinical abilities while still benefiting from the expertise of their tutors. The presence of a supportive tutor during real patient interactions helped to alleviate the stress and anxiety that students often experience in clinical settings, particularly in sensitive fields like gynecology. This supportive relationship was further evidenced by the students’ high satisfaction ratings (Table 1), with many expressing a desire for more opportunities to participate in STCs.

Feedback is an essential component of effective teaching and learning (16–18). Literature suggested that learning is enhanced if feedback is specific (19), and a “move away from apprenticeship to experiential learning” has been advocated (20). STCs can serve this critical function if tutors are trained to give constructive feedback. According to the importance of constructive feedback, anticipatory thinking and ‘mental simulation’ can enhance learning (21). Repeated exposure to supervised patient clerking and examination by more widespread use of STCs would allow learners to develop their mastery, reinforcing Ericsson’s theory of deliberate practice (22) and Bloom’s theory of mastery (23). Such practice and refinement facilitate learning in a practical environment.

In addition to the tutor-student relationship, the STC also facilitated the development of strong student-patient relationships. Unlike simulated patient interactions, which can sometimes feel artificial, the use of real patients in the STC provided students with a more authentic clinical experience. This authenticity is critical for the development of interpersonal skills such as empathy, active listening, and effective communication (24). The positive feedback from patients, who reported high levels of satisfaction with their STC experience (Table 3), indicates that the students were able to establish meaningful connections with their patients. These relationships not only enhanced the students’ learning experience but also contributed to the overall quality of care provided during the consultations.

Male doctors may face some cultural or social barriers during gynecological clinical work, especially when they are still medical students (25). In our study, Improvement in general self-efficiency (Figure 2) and performance (Figure 3) during the consultation were observed in both male and female students.

Furthermore, the relational aspect of the STC extended to peer relationships within the student groups. The collaborative nature of the STC, where students observed and learned from each other’s consultations, fostered a sense of camaraderie and mutual support. This peer learning dynamic is particularly valuable in medical education, where the exchange of ideas and experiences among students can enhance understanding and retention of clinical knowledge (26). The opportunity to observe their peers in action also provided students with additional learning opportunities, as they could critically assess different approaches to patient care and incorporate best practices into their own consultations.

### 4.3 Supporting services

The third dimension, supporting services, is integral to the success of any educational program, particularly in a clinical setting. In the STC, the availability of robust support services ensured that students could focus on their learning without being hindered by logistical challenges. These services encompassed a range of resources, from the physical setup of the clinic to the psychological support provided to students.

The physical environment of the STC was carefully designed to mimic a real clinical setting, providing students with a realistic context in which to apply their skills. Teaching in the presence of real patients in a clinical setting is ideal for medical education (27). There is evidence that patient inclusion contributes to a positive medical learning environment (28). It provides a critical component for students to learn humanistic and professional behaviors (29), especially when doctors who are considered excellent tutors model positive patient care behaviors in clinical work. This environment also included the access to essential medical equipment, private consultation rooms, and comfortable spaces for students to discuss and reflect on their experiences. The importance of a well-equipped learning environment cannot be overstated, as it directly impacts the quality of the educational experience (30). To ensure the safety and comfort of all individuals, it is essential to view the environment of STC as a patient- centered clinic rather than an instructional setting.

In addition to the physical resources, the STC also provided significant psychological support to students. Medical education is inherently stressful, and the transition from theoretical learning to practical application can be daunting for many students (31). Qualified tutors should provide optimal patient care while ensuring a high quality of clinical teaching. Besides maximizing students’ opportunities to consult with real patients, they must maintain patients’ safety and privacy. To preserve the student-patient relationship and create a supportive environment for effective feedback, in-depth discussions about differential diagnosis or management should be conducted after the patients have departed (32). This psychological support was further reinforced by the structured nature of the STC, which included regular debriefing sessions where students could discuss their experiences and receive encouragement from their peers and tutors.

The supporting services also extended to the administrative aspects of the STC. The careful selection of patients, the scheduling of consultations, and the coordination of tutor involvement were all managed to ensure that the STC operated smoothly and efficiently. Real patient-based form of education introduces students to the supervised and structured clinical environment, providing opportunities to participate in medical interviews, humanistic qualities, history taking, physical examination, clinical reasoning, and organization (33). Emergent and vague cases are not appropriate for STCs. Common and frequently occurring gynecological diseases, such as endometriosis or leiomyoma, are ideal for STCs. Moreover, informed consent about recording the consultation process is needed.

### 4.4 Healthcare during the STC

Patient involvement allows for greater patient inclusion in making decisions, encourages efficiency in history presentations and evaluations (34, 35). Previous reports show that patients also benefit from clinical teaching experiences with better understanding of their disease, more compassion and respect from the medical team, and increased inclusion during the medical care (36).

Patient satisfaction and consultation outcomes are always the most significant concerns during STCs. This study seeks to find the optimal balance between student-centered education and patient-centered care (Figure 4). Moreover, we found that patients received appropriate treatment and effective counseling during the STC and were satisfied with the overall experience. Patients have dual requirements for participating in the consultations: the need for reassurance of their medical conditions and hope to help students (37). All participating patients share the wish to help students learn, indicating a high potential for mutually beneficial student-patient relationships during STCs (38). Learning is founded on a reciprocal relationship between patients and students, leading to patients actively engaging in the learning process, which they perceive as a collaborative effort (39). Studies indicate that patients are generally satisfied with the care delivered by medical students and uphold a favorable perception of their interactions with these students (40, 41). The patient’s role includes a secondary benefit of student-led consultations: the sense of contributing to the education of future doctors (42). In the present study, patients involved in the STC reported high satisfaction (Table 3), maybe even higher than that in routine clinics. Since students always “asked more non-critical questions,” which made the patients feel taken more seriously. Patients were also pleased that two medical staff (including at least one certificated gynecologist) were dealing with their problems, making them feel safer and that the consultation was more effective.

### 4.5 Implications for practice and future research

This study has important implications for clinical education. The STC model, with its emphasis on goal direction, relationships, and supporting services, serves as a guide for developing effective medical education. Integrating these three dimensions into the design and implementation of clinical teaching programs may maximize learning outcomes for medical students.

For practice, our findings suggest that medical schools should consider incorporating STCs into their curricula. This model, with its benefits such as improving clinical competency, enhancing interpersonal communication, and boosting student satisfaction, might be a valuable addition to traditional clinical training methods. However, the successful adoption of STCs requires significant investment in well-trained tutors, equipped clinical spaces, and robust administrative systems. For example, detailed and standardized mini-CEX scoring indicators based on teaching objectives should be set, which tutors can use in subsequent STC after training.

Future research should explore the long-term impacts of STCs on student outcomes. While this study demonstrated improvements in self-efficacy and clinical skills, further research is needed to determine if these gains translate into professional practice. Longitudinal studies that track students from their time in the STC through to their early years of practice would provide valuable insights into the effectiveness of this educational model. Additionally, research could explore the potential for adapting the STC model to other areas of medical education, such as surgical training or primary care, where the principles of goal direction, relationships, and supporting services are equally relevant.

### 4.6 Limitations

The study’s observation period was short, focusing on the immediate effects of STC training. Long-term impacts on professional development and clinical skills acquisition may not be fully captured in this timeframe. Future research should consider longitudinal studies to evaluate the enduring effects of STC on students’ career trajectories and clinical competence. Additionally, although the mini-CEX was employed as an assessment methodology it is by nature subjective and based on tutor’s observations. Variability in grading standards among tutors may affect the consistency and reliability of the results.

## 5 Conclusion

This study has demonstrated the STC model at a tertiary teaching hospital., when designed with a focus on goal direction, relationships, and supporting services, provides a highly effective framework for medical education. The STC not only enhances students’ clinical competencies but also fosters the development of critical interpersonal skills and provides the necessary support for a positive learning experience. The findings of this study suggest that the STC model could be considered to further integrated into medical curricula, with continued research and investment to optimize its implementation. In the future of medical education, the STC offers a promising approach to preparing healthcare professionals for the challenges of clinical practice.

## Data Availability

The original contributions presented in the study are included in the article/Supplementary material, further inquiries can be directed to the corresponding author.
