# Supplementary material for: From shadowing to active learning: exploring the impact of supervised teaching clinics on gynecology education
Source: Front Med (Lausanne). 2025 Jan 7;11:1498393. doi: 10.3389/fmed.2024.1498393 (PMC11747641; doi:10.3389/fmed.2024.1498393)
Supplement: Supplementary file 1 [file Table_1.DOCX]

**Supplementary Table 1 The demographic information of students participating in the study**

| **Students** | **Ctrl group (n=80)** | **STC group (n=64)** |
| --- | --- | --- |
| **Age (yr)** | 23.49±0.50 | 23.38±0.49 |
| **Gender** |  |  |
| - **Male** | 35 | 30 |
| - **Female** | 45 | 34 |

**Supplementary Table 2 The demographic information of patients participating in STC**

| **Patients** |  |
| --- | --- |
| **Age (yr)** | 38.78±9.69 |
| **Gestation** | 2.53±1.80 |
| **Parturition** | 1.19±0.86 |
| **Gynecological disease (n=97,100%)** |  |
| - **Endometriosis** | 18 (18.56%) |
| - **Leiomyoma** | 17 (17.53%) |
| - **Adenomyosis** | 12 12.37(%) |
| - **Adnexal masses** | 11 (11.34%) |
| - **Cervix disease** | 9 (9.28%) |
| - **Abnormal uterine bleeding** | 9 (9.28%) |
| - **Early pregnancy** | 7 (7.22%) |
| - **Others** | 16 (16.50%) |
